# CD47 Promotes Tumor Invasion and Metastasis in Non-small Cell Lung Cancer

**DOI:** 10.1038/srep29719

**Published:** 2016-07-14

**Authors:** Hui Zhao, Jianxin Wang, Xiaodan Kong, Encheng Li, Yuanbin Liu, Xiaohui Du, Zhijie Kang, Ying Tang, Yanbin Kuang, Zhihui Yang, Youwen Zhou, Qi Wang

**Affiliations:** 1 Department of Respiratory Medicine, The Second Affiliated Hospital, Dalian Medical University, Dalian, China; 2 Department of Rheumatology, The Second Affiliated Hospital, Dalian Medical University, Dalian, China; 3 Key Laboratory of Laboratory Medicine, Ministry of Education, Zhejiang Provincial Key Laboratory of Medical Genetics, Wenzhou Medical University, Wenzhou, China; 4 Scientific Research Center, The Second Affiliated Hospital, Dalian Medical University, Dalian, China; 5 Department of Hematology Medicine, The Second Affiliated Hospital, Dalian Medical University, Dalian, China; 6 Department of Pathology Medicine, The Second Affiliated Hospital, Dalian Medical University, Dalian, China; 7 Molecular Medicine Lab, University of British Columbia, Vancouver, BC, Canada

## Abstract

CD47 is overexpressed in many human cancers, its level positively correlates with tumor invasion and metastasis. However, it is largely unknown whether CD47 overexpression drives metastasis and how CD47 lead to tumor metastasis in non-small cell lung cancer (NSCLC). In this study, we analyzed NSCLC specimens and cell lines, and revealed that CD47 is expressed at a higher level than in tumor-free control samples. Furthermore, increased CD47 expression correlated with clinical staging, lymph node metastasis and distant metastasis. In order to understand the molecular mechanisms underlying CD47 functions, we applied both gain-of-function and loss-of-function approaches in cell lines. The siRNA-mediated downregulation of CD47 inhibited cell invasion and metastasis *in vitro*, while the overexpression of CD47 by plasmid transfection generated opposite effects. *In vivo,* CD47-specific shRNA significantly reduced tumor growth and metastasis. On the molecular level, the expression of CD47 correlated with that of Cdc42, both in cell lines and NSCLC specimens. The inhibition of Cdc42 attenuates the invasion and metastasis of CD47-overexpressing cells. These results indicate that Cdc42 is a downstream mediator of CD47-promoted metastasis. Our findings provide first evidence that CD47 is an adverse prognostic factor for disease progression and metastasis, and a promising therapeutic target for NSCLC.

Lung cancer is the most common malignancy worldwide, and the leading cause of cancer-related death. The estimated incidence of lung cancer was 1.8 million in 2012, representing 12.9% of all newly diagnosed cancers^1^. Among these two major types of lung cancers, non-small cell lung cancer (NSCLC) accounts for approximately 85% of all cases; and in clinic, most NSCLC patients present with locally advanced or metastatic diseases^2^,^3^. Limited by effective chemotherapeutic agents, the overall 5-year survival of NSCLC marginally increased over the last decade (from 15.7% to 17.4%)^4^. Therefore, it is imperative to develop effective and safe therapies that target the aggressive progression and metastasis of NSCLC.

CD47 is a transmembrane glycoprotein that is ubiquitously expressed in normal tissues and mediates a “self/don’t-eat-me” signal on normal cells by inhibiting macrophage phagocytosis through its interaction with macrophage signal regulatory protein alpha (SIRPα)^5^,^6^. The enhanced expression of CD47 has also been reported in various malignancies including leukemia^7^,^8^, lymphoma^9^, multiple myeloma^10^ and solid tumors such as breast^11^, colon^12^, hepatocellular carcinoma^13^, and melanoma^14^. It has been demonstrated that CD47 expression facilitates the immunological evasion of tumor cells^15^, implying the therapeutic potential of targeting CD47 in various malignancies^16^. However, few are known on the expression and functional significance of CD47 in NSCLC. On account of the molecular mediator of CD47, cell division control protein 42 (Cdc42) has been shown to be activated downstream of CD47 to promote neurite and filopodium formation^17^,^18^. Cdc42 is a member of the Rho family of small GTPases, is identified as an important regulator of metastasis^19^, and is overexpressed in a number of human cancers^20^. Subsequent studies have demonstrated that CD47 and its downstream signaling effector, Cdc42, can facilitate the formation of protrusions of the lamellipodia and filopodia to impact cell mobility^17^,^21^. Nevertheless, whether Cdc42 is a clinically relevant downstream target of CD47 with a critical role in promoting invasion and metastasis in NSCLC needs to be further explored.

In the present study, we investigated the expression of CD47 in NSCLC in clinic, as well as the experimental settings; and explored the therapeutic potential of targeting CD47 with small interfering RNA (siRNA) and the underlying regulatory mechanisms, with specific focus on Cdc42, both *in vitro* and *in vivo.*

## Results

### CD47 is overexpressed in primary human NSCLC tissues and NSCLC cell lines

**Figure 1:**
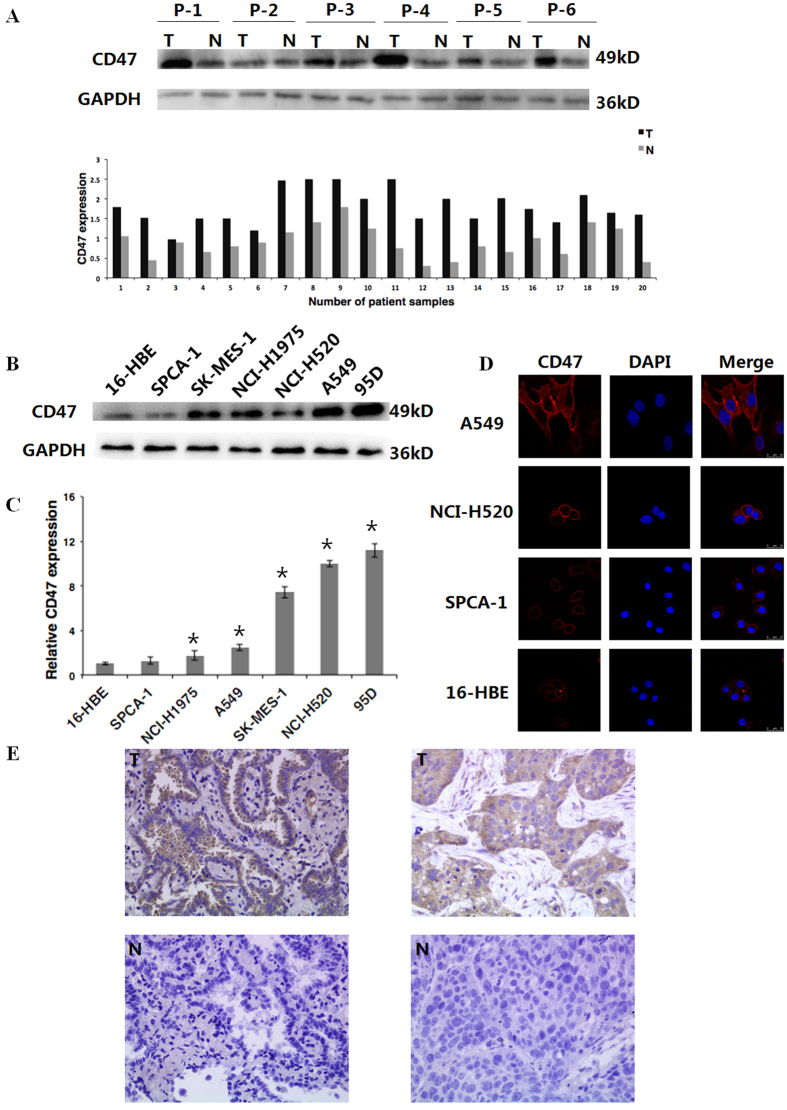
CD47 is overexpressed in NSCLC tissues and NSCLC cell lines. (**A**) Representative western blotting analysis of the expression of CD47 protein in paired NSCLC tissues (T) and adjacent non-tumor tissues (N), upper panel. GAPDH was used as a loading control. P, patient; Lowe panel, the quantification of CD47 expression (calculated as the ratio of CD47 signal intensity to GAPDH signal intensity) in 20 NSCLC tissues and matched adjacent non-tumor tissues. (**B**) Western blot analysis of CD47 expression in indicated NSCLC and normal bronchial epithelial cell lines. GAPDH was used as a loading control. (**C**) RT-qPCR on CD47 steady-state mRNA levels in the indicated NSCLC and normal bronchial epithelial cell lines. *P < 0.05, compared to 16-HBE cells. (**D**) Immunofluorescent staining for CD47 (red) in A549, NCI-H520, SPCA-1 and 16HBE cells. Nuclei were stained with DAPI (blue). (E) Representative immunohistochemical staining of CD47 (brown signal) in human NSCLC tissues (T) and matched adjacent non-tumor tissues (N) from a patient with squamous cell carcinoma (left) and a patient with adenocarcinoma (right); Magnification, × 400.

CD47 overexpression has been reported in many human cancers; however, its expression in NSCLC remains unclear. In order to address this question, western blot analyses were performed on 20 pairs of matched NSCLC tumor tissues and adjacent non-tumor tissues. Results revealed that the expression of CD47 was significantly higher in cancer tissues than in matched adjacent non-tumor tissues (Fig. 1A). Furthermore, CD47 expression was measured on protein (Fig. 1B) and steady-state mRNA levels (Fig. 1C) in a series of NSCLC cell lines (NCI-H1975, NCI-H520, SPCA-1, SK-MES-1, A549 and 95D) and in normal human bronchial epithelial cell line 16-HBE. The analyses revealed that all cancer cell lines, except SPCA-1, expressed significantly higher levels of CD47 protein and mRNA than 16HBE cells. Consistently, the immunofluorescence staining of CD47 on these cells revealed that a strong membrane staining of CD47 in A549 cells, intermediate staining in NCI-H520 cells and minimal staining in SPCA-1 and 16-HBE cells (Fig. 1D).

### Increased expression of CD47 correlates with metastasis and progression in human NSCLC

The overexpression of CD47 in human NSCLC tumor tissues and cell lines suggest that it might be associated with disease progression. In order to test this possibility, CD47 expression was examined in 80 human NSCLC tissues by IHC, expression levels were quantified using the staining index, and the relationship between CD47 expression and the different clinicopathologic features of these NSCLC patients were analyzed. IHC staining revealed a strong CD47-positive staining in the membrane and cytoplasm of tumor cells, compared with that in adjacent normal lung cells, which had a weak stain or no staining (Fig. 1E). Based on staining index scores, the cohort of 80 NSCLC patients was divided into low and high CD47 expression groups. As shown in Table 1, higher CD47 expression levels positively and significantly correlated with the T classification, clinical staging, lymph node metastasis and distant metastasis of NSCLC; but not with age, gender, differentiation status or histological subtypes.

### CD47 critically controls NSCLC cell migration/invasion, but exerts minimal effects on cell viability *in vitro*.

**Figure 2:**
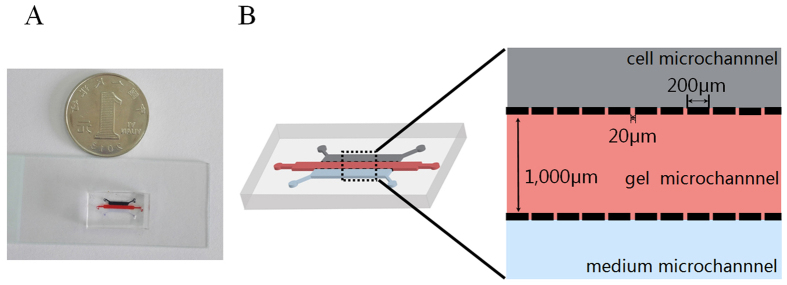
Photograph (**A**) and illustration (**B**) of the home-made microfluidic chip. The chip comprises two side micro-channels sandwiching a gel micro-channel with two rows of micro-gaps. The gel micro-channel was filled with Matrigel (red), and the cell micro-channel and medium micro-channel were used for plating the cells (black) and the chemoattractant (20% FBS) (blue), respectively.

**Figure 3:**
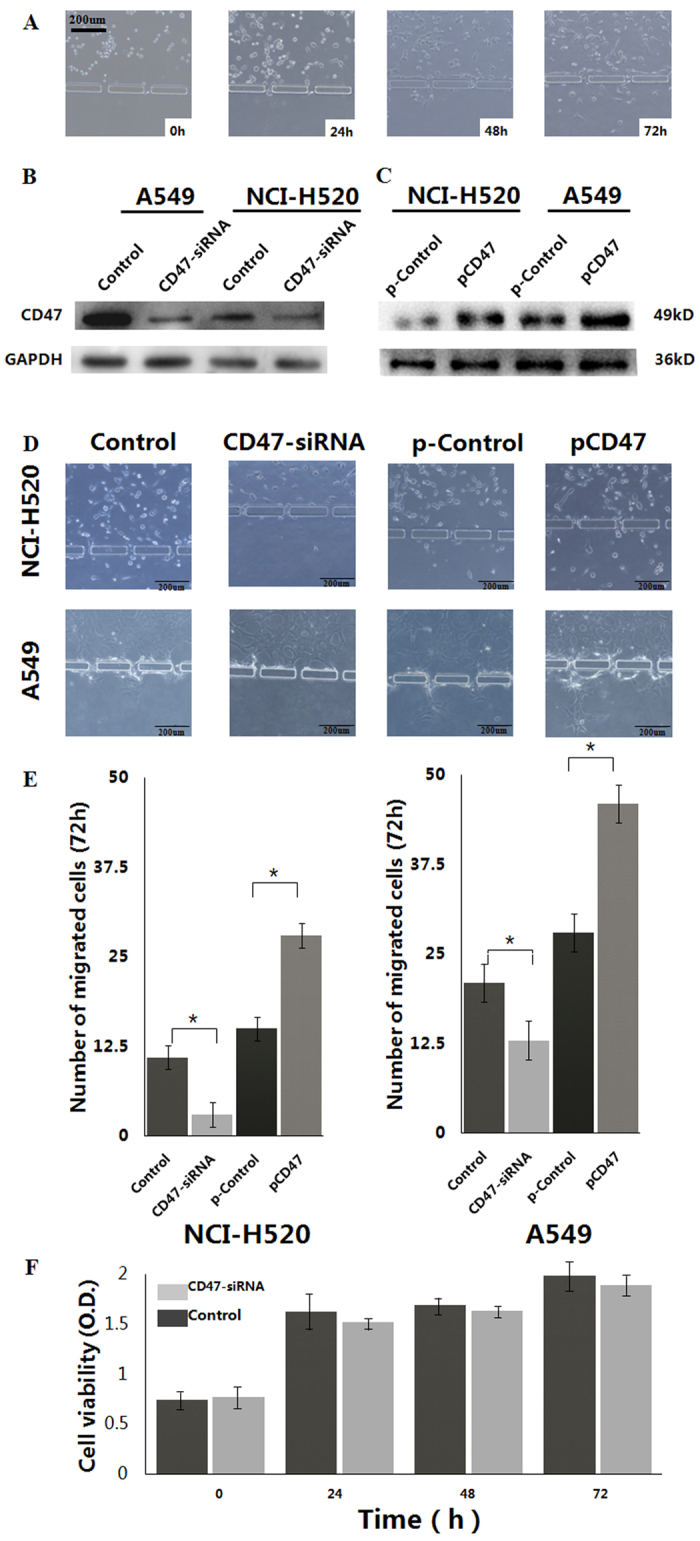
CD47 critically controls the migration/invasion of NSCLC cells *in vitro*. (**A**) Migration/invasion of HCI-H520 cells to the gel micro-channel at different time points. (**B**) Western blotting analysis of CD47 protein level in A549 and HCI-H520 cells transfected with control siRNA (control) or siRNA against CD47 (CD47-siRNA), respectively. (**C**) Western blotting analysis of CD47 protein level in A549 and HCI-H520 cells transfected with control vector (pControl) or pcDNA3.1-3xFlag-CD47 (pCD47), respectively. GAPDH was used as a loading control. (**D**) Representative images of A549 and HCI-H520 cells were transfected with CD47-siRNA and pcDNA3.1-3xFlag-CD47, respectively. (**E**) Quantification of migrated A549 and HCI-H520 cells in comparing the CD47-siRNA treated group or pcDNA3.1-3xFlag-CD47 treated group with the control groups; *P < 0.05. (**F**) MTT assay for cell viability at different time points after siRNA transfection.

In order to characterize the functional significance of CD47 in NSCLC cells, a homemade microfluidic chip (Fig. 2) was constructed to measure the migration and invasion of target cells from a liquid environment, through 20-μm pores and into the Matrigel. As shown in Fig. 3A, using 20% FBS as a chemoattractant, NCI-H520 cells progressively exited from the cell micro-channel through the micro-gaps, and invaded into the gel micro-channel over 72 hours. Next, A549 and NCI-H520 cells were transfected with siRNA, specifically targeting CD47 (CD47-siRNA); and revealed that CD47-siRNA dramatically reduced the endogenous expression of CD47 (Fig. 3B). By comparing with the migration/invasion of control siRNA-transfected cells, we found that CD47 knockdown significantly suppressed A549 and NCI-H520 cell migration/invasion (Fig. 3D,E, *P* < 0.05). Furthermore, the overexpression of CD47 in A549 and NCI-H520 cells by transient transfection of pcDNA3.1-3xFlag-CD47 was confirmed by western blot (Fig. 3C). The migration/invasion of pcDNA3.1-3xFlag-CD47-transfected cells significantly increased migration and invasion abilities, compared to that of control cells (Fig. 3D,E, *P* < 0.05). These results indicate that the migration and invasion of NSCLC cells were significantly increased by the exogenous overexpression of CD47, while the downregulation of CD47 by small interfering RNA molecules suppressed the metastatic potential of NSCLC cells. Thus, CD47 activation appears to be tightly correlated with cell migration and invasion ability; hence, CD47 might be an important regulator of migration and invasion in lung cancer cells. In contrast, when focusing on cell viability, the knockdown of endogenous CD47 did not reduce cell viability over 72 hours after siRNA transfection (Fig. 3F); indicating that the action of CD47-siRNA in reducing A549 migration/invasion was probably not due to the significant cytotoxic effect of CD47 knockdown.

### Cdc42 is a downstream mediator for CD47-induced migration in NSCLC

**Figure 4:**
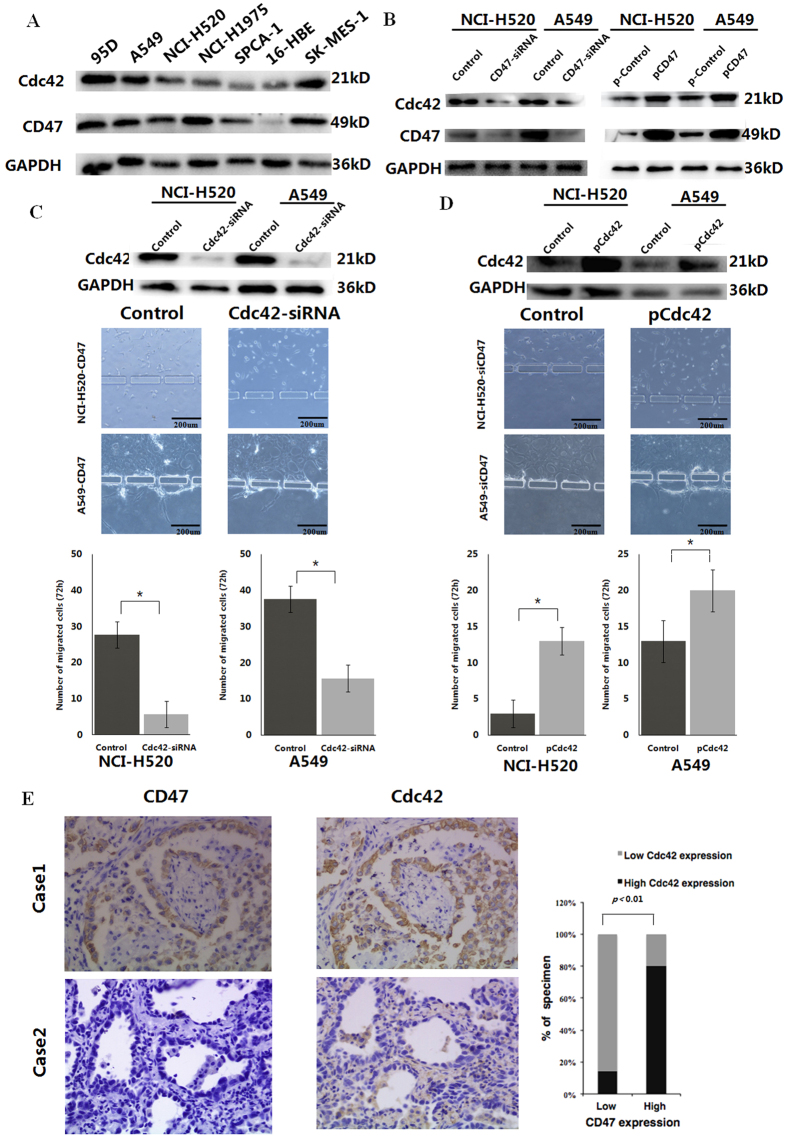
CD47 expression correlates with Cdc42 expression in NSCLC. (**A**) Western blotting analysis on CD47 and Cdc42 protein expression in indicated NSCLC cell lines. (**B**) Western blotting analysis of CD47 and Cdc42 expression in A549 and NCI-H520 cells transfected with CD47-siRNA *vs.* control siRNA (left) and A549 and NCI-H520 cells transfected with pcDNA3.1-3xFlag-CD47 *vs.* control vector (right). (**C**) Representative images and quantification of invading CD47-expressing A549 and NCI-H520 cells transfected with control siRNA or Cdc42-siRNA; *P < 0.05. (**D**) Cdc42 overexpression rescued the effects of CD47 siRNA on A549 and NCI-H520 cell migration and invasion; *P < 0.05. (**E**) Representative IHC images and quantification of NSCLC specimens show the correlation between CD47 expression and Cdc42 expression. Error bars represent the means of three independent experiments.

In order to understand the molecular mechanism underlying CD47-induced migration/invasion of NSCLC cells, we focused on Cdc42, a pleotropic protein known to be overexpressed in NSCLC^22^, and correlated this with NSCLC cell colonization and metastasis formation^11^. Recent studies have shown that Cdc42 is activated downstream of CD47 and regulates melanoma cell migration^17^. Therefore, we tested whether Cdc42 may also be a downstream mediator of CD47-induced tumor invasion in NSCLC. First, the expression level of CD47 was found to be correlated with that of Cdc42 in NSCLC cell lines (Fig. 4A). Second, the knockdown of endogenous CD47 in A549 and NCI-H520 cells with siRNA significantly reduced Cdc42 levels in these cells, while ectopically expressing CD47 in A549 and NCI-H520 cells significantly boosted Cdc42 levels (Fig. 4B); suggesting the control by CD47 on Cdc42 expression. Functionally, in A549 and NCI-H520 cells that ectopically expressed CD47, the knockdown of Cdc42 levels with siRNA significantly reduced cell migration/invasion (Fig. 4C). Conversely, when we elevated the expression of Cdc42 in CD47-siRNA-transfected A549 or NCI-H520 cells, the reduced migration/invasion was significantly boosted (Fig. 4D), suggesting the essential role of Cdc42 in CD47-induced NSCLC migration/invasion. Lastly, the clinical correlation between CD47 and Cdc42 expression was examined in 80 NSCLC patients with advanced diseases. As shown in Fig. 4E, strong co-staining of both proteins from sequential sections could be detected in tumor samples with high CD47 expression, while minimal staining of Cdc42 was detected in those with low CD47 expression. Statistical analysis revealed that 80.4% of NSCLC specimens with high CD47 expression also exhibited strong Cdc42 staining signals, and 85.3% of the tumor samples with low CD47 expression displayed low or undetectable Cdc42 signals (*X*^*2*^ = 33.87, *P* < 0.01). Therefore, CD47 most likely controls Cdc42 expression in NSCLC, and Cdc42 in return mediates the CD47-induced migration/invasion phenotype of these cells.

### CD47 contributes to NSCLC tumor growth and metastasis *in vivo*

**Figure 5:**
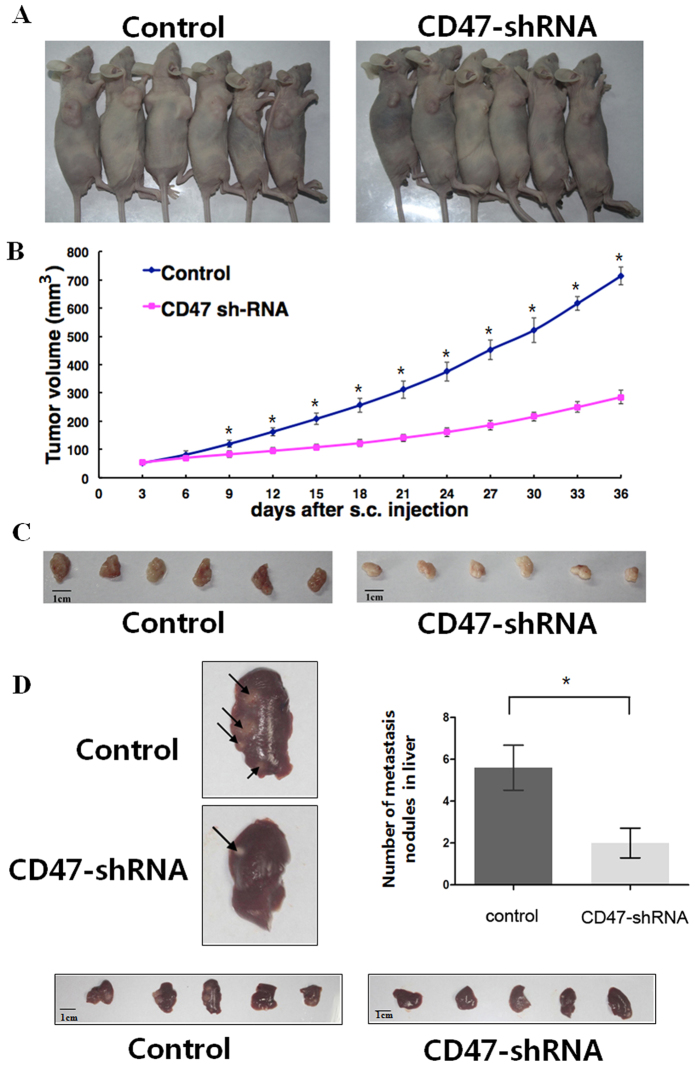
CD47 inhibits the growth and metastasis of NSCLC xenografts *in vivo*. (**A**) Photographs of mice bearing subcutaneous tumors from A549 cells stably transfected with control shRNA (left) and CD47-shRNA (right) on day 36 after cell injection. (**B**) Tumor growth curve over time; *P < 0.05. (**C**) Photographs of isolated tumors on day 36 after tumor cell injection. (**D**) Representative pictures of mouse liver are presented. Arrows indicate metastatic nodules on liver surface. The number of metastatic nodules in mice (*n* = 6/group) significantly decreased in the CD47-shRNA group (*P < 0.05).

In order to understand the functional significance of CD47 in NSCLC tumor development, xenograft tumors were established using A549 cells stably transfected with either CD47-shRNA or control shRNA, and followed tumor growth over time. As shown in Fig. 5A, the knockdown of CD47 in A549 cells significantly inhibited tumor growth *in vivo,* leading to much smaller tumors at 36 days after A549 injection. Mean tumor volume by day 36 was 285.2 ± 23.7 mm^3^ in the CD47-shRNA group and 714.1 ± 31.8 mm^3^ in the control shRNA group (Fig. 5B,C, *P* < 0.05).

In order to assess the effect of CD47 on *in vivo* tumor metastasis, A549 transfected with control shRNA or CD47-shRNA was injected through the tail vein and quantified the number of metastatic foci in the liver after four weeks^23^. As shown in Fig. 5D, mice injected with CD47-shRNA cells displayed a significantly lower number of metastatic nodules in the liver when compared to the corresponding control group (*P* < 0.05).

## Discussion

This study is the first to reveal that CD47 was overexpressed in NSCLC tumor tissues and cell lines, compared to matching adjacent non-tumor tissues and normal bronchial epithelial cells, respectively. The overexpression of CD47 significantly correlated with T classification, clinical staging, lymph node metastasis and distant metastasis. On the cellular level, CD47 critically controlled the migration/invasion behavior of NSCLC cells, which was mediated through the regulation of Cdc42 expression. Consistently, targeting CD47 significantly inhibited tumor growth and metastasis *in vivo.* These results revealed that CD47 is an important prognostic marker and a potential therapeutic target for NSCLC.

Previous studies have indicated that CD47 is a novel prognostic biomarker for several malignancies^8^,^9^,^24^,^25^,^26^. For example, Baccelli *et al*. reported that the presence of luminal breast cancer metastasis-initiating cells (MICs) with the characteristic EPCAM^+^CD44^+^CD47^+^MET^+/−^ phenotype correlates with lower overall survival and increased number of metastatic sites^5^. Furthermore, circulating tumor cells (CTC) positive for CD47 expression are responsible for tumor relapse and metastasis in breast cancer patients^27^, while constitutive CD47 upregulation is essential for non-Hodgkin lymphoma immunotolerance and dissemination^9^. In colorectal cancer, the upregulation of the *CD47* gene, as a potential immune-escape mechanism, is related to distant metastasis^12^. Consistent with these reports, siRNA targeting CD47 effectively inhibited melanoma growth and lung metastasis^14^. Furthermore, blocking CD47 signaling inhibits tumor growth and metastasis, proving its benefits for the treatment of several types of cancers^28^,^29^. In this study, we provided novel evidence for the overexpression of CD47, as well as its biological significance and functional consequence in NSCLC, using both human tumor tissues and cancer cell lines.

In order to investigate the migration and invasion behavior of NSCLC cells, we constructed a three-dimensional (3D) microfluidic chip-based *in vitro* assay system. This system overcomes some of the limitations associated with conventional 2D experimental systems (such as wound assay and Boyden chamber assay), including cell migration that occurs over a 2-dimensional (2D) substrate. Furthermore, these systems do not represent environments such as those found *in vivo*, and its quantification is somewhat arbitrary and does not reflect real-time changes^30^,^31^,^32^,^33^. In the 3D microfluidic chip, cancer cells are cultured in suspension within the medium channel, mimicking the *in vivo* state of these cells in the circulation^34^. The presence of micro-gaps in micro-columns that separating these two medium channels from the central gel channel allows the diffusion of chemoattractants from serum into the Matrigel, as well as the migration/invasion of tumor cells in response to the gradient of these chemoattractants. Although other studies have included multiple cell types into the gel channel to better mimick an *in vivo* vascular endothelium and perivascular microenvironment^31^, our model provides a proof-of-principle method to investigate the extravasation behavior of tumor cells. Cell migration/invasion was quantified on those moving from the cell micro-channel, through the micro-gaps, and into the gel micro-channel. Using this chip, we studied the effect of altering CD47 expression in NSCLC cells on cell migration. For A549 and NCI-H520 cells, the knockdown of CD47 with siRNA significantly inhibited cell migration/invasion. Meanwhile, the ectopic transfection of CD47 into A549 and NCI-H520 cell significantly enhanced cell migration/invasion, indicating that CD47 is both necessary and sufficient for regulating the NSCLC migration/invasion phenotype. Furthermore, we did not detect any direct cytotoxicity of CD47 siRNA on NSCLC cells by MTT assay, suggesting that benefits from CD47 siRNA at least *in vitro* was not due to the direct toxicity on tumor cells, but rather from the inhibition of cell migration/invasion. Furthermore, we found that the downregulation of CD47 in NSCLC cells *in vivo* significantly inhibited tumor growth and metastasis; suggesting that CD47 critically controls tumor cell growth and metastasis *in vivo*, and supporting the therapeutic potential of targeting CD47 for NSCLC treatment.

Although many recent studies^16^,^35^ have focused on the “don’t eat me” signal mediated through the interaction between CD47 on cancer cells and SIRPα on macrophages, additional mechanisms may also contribute to the anti-tumor effects of CD47 siRNA^36^. Cdc42 has been shown to be activated downstream of CD47^18^ and facilitate the formation of protrusions of lamellipodia and filopodia to impact cell mobility^17^,^21^. Cdc42 overexpression was also detected in lung cancer^22^ and served as a disease marker and prognosis predictor^37^,^38^. Consistently, we demonstrated the overexpression of Cdc42 in NSCLC tissues, compared with normal tissues (data not shown). Furthermore, we identified the significant correlation between the expression levels of CD47 and Cdc42, both in NSCLC tissues and cell lines. By using both gain-of-function and loss-of-function approaches in different NSCLC cell lines, we demonstrated that the overexpression of CD47 led to increased Cdc42 expression; while the knockdown of CD47 had an opposite effect. Nevertheless, the precise molecular mechanism underlying the CD47-induced upregulation of Cdc42 remains unknown. CD47, as an integrin-associated protein, regulates multiple cellular behaviors through its interaction with integrin, in addition to other ligands, including cell adhesion, migration, and invasion^39^. Integrin may also signal through 14-3-3zeta to activate Cdc42 and Rac1, and modulate cytoskeletal re-organization^40^. Thus, integrin may link the activation between CD47 and Cdc42. Further studies are required in order to identify the specific integrin member, since integrin is a family of cell surface receptors that contain 24 heterodimers from the 18α-subunits and 8β-subunits^41^. It should be noted that, in this study, we also explored the relationship between the expression of CD47 and several markers involved in epithelial-mesenchymal transition (EMT). This is critical for the metastasis of NSCLC, including E-cadherin, N-cadherin, Snail-1 and Snail-2. Furthermore, no relevance was identified (Supplemental Fig. 1); suggesting that the pro-migration/invasion activity of CD47 could not be achieved through regulation in the EMT program. Meanwhile, the role of EMT in metastasis remains under debate. Gao *et al*. found that lung metastases mainly consist of non-EMT tumor cells that maintain their epithelial phenotype. This study suggests that EMT is not required for lung metastasis^42^.

In summary, we revealed that CD47 is overexpressed in NSCLC, and that it is correlated with advanced tumor progression and more aggressive metastasis. More remarkably, our findings suggest that the knockdown of CD47 significantly reduced cell migration *in vitro*, and inhibited NSCLC tumor growth and metastasis *in vivo*. Furthermore, Cdc42 is a downstream mediator for CD47-induced NSCLC migration/invasion. Therefore, our study provides a promising molecular target for designing rational therapeutic modalities to control NSCLC.

## Materials and Methods

### Patients and tissue samples

From January 2006 to May 2015, 80 patients who underwent surgical resections for NSCLC at the Second Affiliated Hospital of Dalian Medical University (Dalian, China) were recruited into this study. Clinical characteristics and pathological information of these patients were obtained from their medical records. During the surgery, at least two tissue samples were obtained from each patient: tumor tissues (T) from the center of the primary cancer, and adjacent non-tumor tissues (ANT) approximately 3 cm from the tumor periphery. All tissue samples were prepared into formalin-fixed paraffin-embedded sections (FFPE, 4 μm) for later analysis by immunohistochemistry (IHC). In addition, for the last 20 consecutive patients, an extra pair of T and ANT samples was obtained from each patient, which was snapped-frozen in liquid nitrogen before storage at −80 °C for future western blotting analysis. The experimental protocol was approved by the Ethics Review Committee of the Second Hospital of Dalian Medical University, and this study was conducted in compliance with ethical and safe research practices involving human subjects or tissues. Informed consent was obtained from all subjects.

### Cell lines

Human NSCLC cell lines A549, NCI-H520, NCI-H1975, 95D, SK-MES-1 and SPCA-1, as well as normal human bronchial epithelial cell line 16-HBE, were obtained from the Cell Bank of Type Culture Collection of Chinese Academy of Sciences (Shanghai, China). These were cultured in recommended medium at 37 °C in a humidified atmosphere of 20% O_2_ and 5% CO_2_. All experiments were performed using exponentially growing cells.

### Western blotting

For western blotting, cells or tissues were directly lysed in RIPA buffer (Cell Signaling Technology) containing a cocktail of protease inhibitors (Sigma, St. Louis, MO, USA). Total proteins were separated by SDS-PAGE electrophoresis and transferred onto BioTrace nitrocellulose membranes (Pall Corporation, San Diego, CA, USA). Then, membranes were blocked with 5% milk-TBST buffer (TBS plus 0.1% Tween-20) for one hour at room temperature and incubated with anti-CD47 (Abcam, Cambridge, UK), anti-Cdc42 (Abcam), or anti-GAPDH (internal control; Sigma) primary antibodies overnight at 4 °C. The membranes were washed with TBST buffer three times and incubated with corresponding secondary antibodies (anti-rabbit IgG or anti-mouse IgG; Cell Signaling Technology, Danvers, MA, USA) for one hour at room temperature. Protein detection was achieved using a Super Signal West Pico Kit (Thermo Fisher Scientific Inc., Anthem, AZ, USA), followed by quantitative densitometric analysis using Eagle Eye II software (London, England). The expression of a target protein was normalized to that of GAPDH, and each experiment was repeated three times.

### Immunohistochemistry

For IHC analysis on tissue CD47 or Cdc42, FFPE sections were blocked with 1% BSA and incubated with mouse anti-CD47 antibody (Sigma) or mouse anti-Cdc42 antibody (Abcam) overnight at 4 °C. Then, the sections were incubated with goat anti-mouse IgG (Maxim, Fuzhou, China) for one hour. Staining results were scored by two investigators blinded to the clinical data. The staining index was calculated as the product of the proportion of positively stained tumor cells and staining intensity. The former was scored based on the percentage of positively stained tumor cells: 0 (0%), 1 (<10%), 2 (10–35%), 3 (35–70%) and 4 (>70%); while staining intensity was graded as: 0 (no staining), 1 (weak), 2 (moderate) and 3 (strong). Therefore, the staining index has a score between 0 and 12. A staining index score ≥6 indicates a high expression, while a score <6 indicate a low expression.

### Reverse transcription and quantitative real-time PCR (qRT-PCR)

Total RNA was isolated from cells using TRIzol reagent (Life Technologies, Carlsbad, CA, USA), according to manufacturer’s instructions. cDNA was synthesized using TransScript One-Step gDNA Removal and cDNA Synthesis SuperMix (Transgen Biotech, Beijing, China). In order to quantitate the expression of human CD47 mRNA, qRT-PCR was performed using TransStart Top Green qPCRSuperMix (Transgen Biotech). Oligonucleotide primers used for CD47 and GAPDH (internal control) were as follows: CD47, 5′-AGATCCGGTGGTATGGATGAGA-3′ (sense) and 5′-GTCACAATTAAACCAAGGCCAGTAG-3′ (antisense); GAPDH, 5′-GCACCGTCAAGGCTGAGAAC-3′ (sense) and 5′-TGGTGAAGACGCCAGTGGA-3′ (antisense). All samples were run in triplicate for target and internal control genes. Cycle threshold (Ct) values of CD47 cDNA were normalized to GAPDH using the ΔΔCt method^43^.

### Immunofluorescence assay

For immunofluorescence staining, cells were grown on a Glass Bottom Cell Culture Dish (Nest, Wuxi, China) until 50–60% confluence, fixed with 4% paraformaldehyde and permeabilized with 0.3% Triton X-100. After washing three times with cold PBS, cells were incubated with anti-CD47 antibodies (Santa Cruz Biotechnology, Santa Cruz, CA, USA) at 4 °C for one hour, followed by Alexa Fluor 594-labeled secondary antibody (Abcam) for one hour, and counterstained with DAPI (Life Technologies). Images were subsequently captured using a confocal microscope (Leica TCS SP5, Mannheim, Germany) and analyzed using ImageJ software.

### Transient or stable transfection of lung cancer cell lines

As a gain-of-function strategy, A549 and NCI-H520 cells were transfected with pcDNA3.1-3xFlag-CD47, pcDNA3.1-3xFlag-Cdc42, or control vector pcDNA3.1-3xFlag plasmids (Youbio Biological Technology Co., Ltd., Beijing China). As a loss-of-function approach, target cells were transfected with CD47 siRNA, Cdc42 siRNA, or control siRNA (Invitrogen, Carlsbad, CA, USA). All transfections were performed with Lipofectamine 3000 (Invitrogen), according to manufacturer’s instructions. The transfected cells were incubated in a humidified incubator at 37 °C with 5% CO_2_ for 72 hours, and collected for further experiments. For stable transfection, a shRNA-expressing plasmid that contain the CD47-targeting sequence (5′-AAAAGCTACTGGCCTTGGTTTAATTCTCGAGAATTAAACCAAGGCCAGTAGC-3′) or a vector containing a scrambled sequence was transfected into the target cells, followed by selection using 2 μg/mL of puromycin for 48 hours. Stable transfected cells were used for subsequent studies.

### Tetrazolium dye methylthiotetrazole (MTT) assay

In order to assess cell viability, A549 cells were seeded into 96-well plates (10^3^ cells/well) and transfected with 100 nM of anti-CD47 siRNA or control siRNA. After incubation at 37 °C for 24, 48 and 72 hours, respectively, 20 μg/mL of MTT (Promega, Madison, WI, USA) was added into each well; and cells were allowed to further incubate at 37 °C for four hours. Then, culture media was removed from the wells, MTT solubilization solution was added, incubated at 37 °C for 15 minutes, and absorbance was measured at 590 nm with a reference filter of 620 nm.

### Fabrication of the microfluidic chip and *in vitro* cell migration/invasion assay

The microfluidic system was fabricated on a micropatterned polydimethylsiloxane (PDMS, Sylgard 184; Dow Chemical, Midland, MI, USA) chip using the standard soft lithographic method, as modified from a previous study^31^. The system composed of a glass coverslip, one central gel micro-channel (9,000 × 1,000 × 40 μm), and two lateral media micro-channels (6,000 × 1,000 × 40 μm) separated by two arrays of micro-columns (200 × 50 × 40 μm, Fig. 2). Each micro-column contained gaps of 50 × 20 × 40 μm in size. In order to quantify cancer cell extravasation, the Matrigel (BD Biosciences, San Jose, CA, USA) was first introduced into the gel micro-channel and incubated at 37 °C in a CO_2_ incubator for 30 minutes to allow hydrogel formation. Then, 50 μL of tumor cells (1 × 10^5^/mL) in serum-free medium were seeded into the cell micro-channel, and medium containing 20% FBS was placed in the media micro-channel as a chemoattractant. Cell migration/invasion was allowed to proceed at 37 °C in a CO_2_ incubator for 72 hours, after which the chip was visualized under a Nikon ECLIPS Ti inverted microscope (Nikon, Tokyo, Japan), with images acquired using a Nikon DS-Ri2camera (Nikon). Three independent experiments were performed for each condition.

### Animal studies

For the establishment of the xenograft tumor model, 5 × 10^6^ cells of A549 cells stably transfected with either control or CD47 shRNA were subcutaneously implanted into the right axillar of 4–5 week-old BALB/c nude mice (*n* = 6 per group, Dalian Medical University). Tumor formation in nude mice was monitored for 36 days, with the length and width measured every three days. Then, mice were euthanized for tumor excision. Tumor volume was calculated as (1/2 × L × W^2^).

For experimental lung metastasis, A549 cells stably transfected with either control or CD47 shRNA (5 × 10^6 ^cells/mouse) were injected into the 4-week-old BALB/c nude mice through tail veins (n = 6 per group). Four weeks later, the mice were sacrificed, with the liver tissues dissected, fixed in 10% formalin, examined under the microscope for metastatic foci. All procedures involving animals were conducted in compliance with a protocol approved by the Institutional Animal Care and Use Committee of the Second Hospital of Dalian Medical University. The experiments were carried out in accordance with approved guidelines.

### Statistical analysis

All statistical analyses were carried out using SPSS 13.0 statistical software package (Chicago, IL, USA). Data were expressed as mean ± standard deviation (SD). Statistical significance of the differences between experimental groups was determined using Student’s *t*-test or *X*^*2*^-test. *P* < 0.05 was considered statistically significant.

## Additional Information

**How to cite this article**: Zhao, H. *et al*. CD47 Promotes Tumor Invasion and Metastasis in Non-small Cell Lung Cancer. *Sci. Rep.* **6**, 29719; doi: 10.1038/srep29719 (2016).

## Supplementary Material

Supplementary Information

## Figures and Tables

**Table 1:** Correlation between CD47 expression level and clinicopathologic characteristics of 80 NSCLC patients.

Variables	CD47			*P-*value
	All cases (*N* = 80)	Low expression (*n* = 34)	High expression (*n* = 46)	
Age (years)				
60	29	12	17	0.878
>60	51	22	29	
Gender				
Male	35	14	21	0.690
Female	45	20	25	
Histological type				
Squamous cell carcinoma	12	5	7	0.310
Adenocarcinoma	65	29	36	
Other	3	0	3	
T factor				
T1 + T2	51	33	18	0.000
T3 + T4	29	1	28	
Lymph node metastasis				
N0 + N1	64	33	31	0.001
N2 + N3	16	1	15	
Distant metastasis				
M0	59	33	26	0.000
M1	21	1	20	
Clinical stage				
I + II	43	33	10	0.000
III + IV	37	1	36	
Differentiation				
Low	23	10	13	0.910
Moderate + high	57	24	33	
