# Supplementary material for: CD47 Promotes Tumor Invasion and Metastasis in Non-small Cell Lung Cancer
Source: Sci Rep. 2016 Jul 14;6:29719. doi: 10.1038/srep29719 (PMC4944213; doi:10.1038/srep29719)

CD47 Promotes Tumor Invasion and Metastasis in Non-small Cell Lung Cancer

Hui Zhao[1#], Jianxin Wang[2#], Xiaodan Kong[3#], Encheng Li[2] , Yuanbin Liu[4], Xiaohui Du[5], Zhijie Kang[6], Ying Tang[7], Yanbin Kuang[2], Zhihui Yang[2] , Youwen Zhou[8*]and Qi Wang[2*]

Supplemental Figure 1

Expression of EMT markers in A549 cells and NCI-H520 cells transfected with control siRNA (control) or siRNA against CD47 (CD47-siRNA) is shown, respectively.

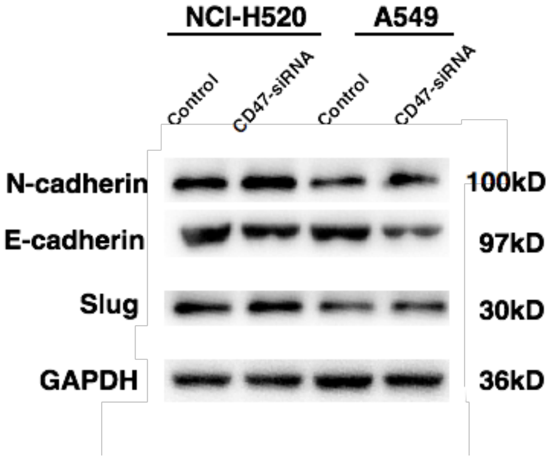

Supplement: Supplementary Information [file srep29719-s1.pdf]
